# Cerebral regional tissue Oxygen Saturation to Guide Oxygen Delivery in preterm neonates during immediate transition after birth (COSGOD III): an investigator-initiated, randomized, multi-center, multi-national, clinical trial on additional cerebral tissue oxygen saturation monitoring combined with defined treatment guidelines versus standard monitoring and treatment as usual in premature infants during immediate transition: study protocol for a randomized controlled trial

**DOI:** 10.1186/s13063-019-3258-y

**Published:** 2019-03-20

**Authors:** Gerhard Pichler, Sigrid Baumgartner, Marlene Biermayr, Eugene Dempsey, Hans Fuchs, Tom G. Goos, Gianluca Lista, Laila Lorenz, Lukasz Karpinski, Souvik Mitra, Lilijana Kornhauser-Cerar, Alexander Avian, Berndt Urlesberger, Georg M. Schmölzer

**Affiliations:** 1 Research Unit for Neonatal Micro- and Macrocirculation, Division of Neonatology, Department of Paediatrics, Medical University of Graz, Auenbruggerplatz 30, 8036 Graz, Austria; 2 Universitätsklinik für Kinder- und Jugendheilkunde Abteilung für Neonatologie, Pädiatrische Intensivmedizin und Neuropädiatrie, Medizinische Universität Wien, Währingergürtel 18-20, Wien, 1090 Austria; 3 Department of Paediatrics II, Neonatology, Medical University of Innsbruck, Christoph-Probst-Platz 1, Innsbruck, 6020 Austria; 4 Infant Centre, University College Cork, Cork University Maternity Hospital, Wilton, Cork, Ireland; 5 Center for Pediatrics, Department of Neonatology, Faculty of Medicine, Medical Center-University of Freiburg, Mathildenstrasse 1, Freiburg, 79106 Germany; 6 Department of Pediatrics, Division of Neonatology, Erasmus MC-Sophia Children’s Hospital, Wytemaweg 80, Rotterdam, 3015 the Netherlands; 7 Department of Biomechanical Engineering, Faculty of Mechanical, Maritime and Materials Engineering, Delft University of Technology, Mekelweg 5, Delft, 2628 The Netherlands; 8 Neonatologia e Terapia Intensiva Neonatale (TIN) Ospedale dei Bambini “V.Buzzi”, Via Castelvetro 32, Milano, 20154 Italy; 9 Department of Neonatology, University Children’s Hospital of Tübingen, Calwerstrasse 7, Tübingen, 72076 Germany; 10 Poznan University of Medical Sciences, Fredry 10, Poznan, 61-701 Poland; 11 Division of Neonatal-Perinatal Medicine, IWK Health Centre, University Avenue 5980, Halifax, B3K 6R8 Nova Scotia Canada; 12 NICU, Division for Perinatology, University Medical Centre Ljubljana, Zaloska cesta 7, Ljubljana, 1000 Slovenia; 13 Institute for Medical Informatics, Statistics and Documentation, Medical University of Graz, Auenbruggerplatz 2, Graz, 8036 Austria; 14 Research Unit for Cerebral Development and Oximetry Research, Division of Neonatology, Department of Paediatrics, Medical University of Graz, Auenbruggerplatz 30, Graz, 8036 Austria; 15 Centre for the Studies of Asphyxia and Resuscitation, Neonatal Research Unit, Royal Alexandra Hospital, Kingsway Avenue 10240, Edmonton, T5H 3V9 Alberta Canada; 16 Department of Pediatrics, University of Alberta, Kingsway Avenue 10240, Edmonton, T5H 3V9 Alberta Canada

**Keywords:** Neonate, Cerebral oxygenation, Immediate transition, Cerebral injury, Mortality

## Abstract

**Background:**

Transition immediately after birth is a complex physiological process. The neonate has to establish sufficient ventilation to ensure significant changes from intra-uterine to extra-uterine circulation. If hypoxia or bradycardia or both occur, as commonly happens during immediate transition in preterm neonates, cerebral hypoxia–ischemia may cause perinatal brain injury.

The primary objective of the COSGOD phase III trial is to investigate whether it is possible to increase survival without cerebral injury in preterm neonates of less than 32 weeks of gestation by targeting cerebral tissue oxygen saturation (crSO_2_) using specified clinical treatment guidelines during the immediate transition period after birth (the first 15 min) in addition to the routine monitoring of arterial oxygen saturation (SpO_2_) and heart rate (HR).

**Methods/Design:**

COSGOD III is an investigator-initiated, randomized, multi-center, multi-national, phase III clinical trial. Inclusion criteria are neonates of less than 32 weeks of gestation, decision to provide full life support, and parental informed consent. Exclusion criteria are severe congenital malformations of brain, heart, lung, or prenatal cerebral injury or a combination of these.

The premature infants will be randomly assigned to study or control groups. Both groups will have a near-infrared spectroscopy (NIRS) device (left frontal), pulse oximeter (right palm/wrist), and electrocardiogram placed immediately after birth. In the study group, the crSO_2_, SpO_2_, and HR readings are visible, and the infant will receive treatment in accordance with defined treatment guidelines. In the control group, only SpO_2_ and HR will be visible, and the infant will receive routine treatment. The intervention period will last for the first 15 min after birth during the immediate transition period and resuscitation. Thereafter, each neonate will be followed up for primary outcome to term date or discharge. The primary outcome is mortality or cerebral injury (or both) defined as any intra-ventricular bleeding or cystic periventricular leukomalacia (or both). Secondary outcomes are neonatal morbidities.

**Discussion:**

crSO_2_ monitoring during immediate transition has been proven to be feasible and improve cerebral oxygenation during immediate transition. The additional monitoring of crSO_2_ with dedicated interventions may improve outcome of preterm neonates as evidenced by increased survival without cerebral injury.

**Trial registration:**

ClinicalTrials.gov Identifier: NCT03166722. Registered March 5, 2017.

## Background

The transition from the intra-uterine to extra-uterine environment is a complex physiological process characterized by major physiological changes in respiratory and hemodynamic functions, which are predominantly initiated by breathing at birth and clamping of the umbilical cord [1]. If hypoxia or bradycardia or both occur, as commonly happens during immediate transition in preterm neonates, cerebral hypoxia–ischemia may cause perinatal brain injury [2–4]. Therefore, protecting the brain from injury is of major importance since up to 10% of infants who survive very preterm birth will develop motor deficits such as cerebral palsy [3] and cognitive deficits [5].

### Monitoring during immediate transition after birth

Routine non-invasive monitoring during immediate transition after birth might include arterial oxygen saturation (SpO_2_) and heart rate (HR) measurement with pulse oximetry/electrocardiogram (ECG), blood pressure, and temperature measurements. Special interest has grown in the use of pulse oximetry and ECG to monitor SpO_2_ and HR during this transitional period [6–10].

However, there is an ongoing debate about the use of supplemental oxygen during neonatal resuscitation. It is still unknown which oxygen concentration is appropriate for preterm infants during immediate transition after birth [10]. In addition, non-invasive monitoring of SpO_2_ does not provide information about adequate oxygen supply to the brain.

Recently, there has been increasing interest in continuous monitoring of cerebral tissue oxygen saturation (crSO_2_) using near-infrared spectroscopy (NIRS) during immediate fetal-to-neonatal transition [2, 11]. In 1992, Peebles et al. reported the first study of NIRS during immediate transition in term neonates and showed a rapid increase of oxygenated hemoglobin and decrease of deoxygenated hemoglobin with initiation of respiration [12]. Further studies showed that crSO_2_ is less affected by mode of delivery (e.g., vaginal delivery versus cesarean section) [13–15] than SpO_2_ and HR. Lower SpO_2_ and HR values have been reported in infants born via cesarean section compared with infants born vaginally [14]. The different behavior of crSO_2_ and SpO_2_ might be due to changes/decreases in cerebral blood flow (CBF) [16]. Differences in arterial oxygen content of the blood or shunting through the patent ductus arteriosus (or both) are most likely the reason for CBF changes/differences [16, 17]. In addition, there is increasing evidence that cerebral tissue oxygenation can be modified by resuscitation interventions in preterm infants [18–21]. It has been demonstrated that preterm infants who need respiratory support showed significantly lower crSO_2_ values compared with those who do not [19].

Therefore, as the brain is the most vulnerable organ of the infant, to monitor cerebral oxygenation in a non-invasive way is a potentially useful to guide supplemental oxygen and respiratory support in neonates. In a two-center prospective observational case control study, we demonstrated that neonates developing an intra-ventricular hemorrhage (IVH) during the first week after birth showed lower crSO_2_ values already during immediate transition compared with neonates without IVH [22].

### COSGOD phase I/II trial

Based on the findings in the observational studies [18–22], preterm neonates of less than 34 weeks of gestation were enrolled in a prospective randomized controlled pilot feasibility study at two tertiary-level neonatal intensive care units (Graz, Austria and Edmonton, Canada) [23]. In an NIRS-visible group, crSO_2_ monitoring in addition to routine monitoring with pulse oximetry and ECG was used to guide respiratory and supplemental oxygen support during the first 15 min after birth. In the NIRS-not-visible group, routine monitoring which consisted of pulse oximetry and ECG was used. The primary outcomes were burden of cerebral hypoxia (<10th centile) or hyperoxia (>90th centile) measured in percentage minutes crSO_2_ during the first 15 min after birth. In the NIRS-visible group, the burden of cerebral hypoxia was halved with a relative reduction of 55.4% (95% confidence interval 37.6–73.2%; *P* = 0.028) [22].

### Assessment of brain injury

Cerebral ultrasonography is a valuable screening tool to determine significant brain injury like IVH grade 1–3 (+ periventricular hemorrhage), cerebellar hemorrhage, and periventricular leukomalacia (PVL) grade 1–3 when conducted regularly over the first weeks of life in preterm infants [24]. Magnetic resonance imaging (MRI) scans of the brain offer even more precise information [25, 26]. However, ultrasound abnormalities in low-gestational-age neonates are already strongly associated with impaired psychomotor and mental development. Children without cranial ultrasound abnormality had the lowest probability of delayed psychomotor or mental development [27].

### Trial objectives

Based on the findings in the observational studies and COSGOD phase I/II trial, the objective of the present clinical trial is to monitor crSO_2_ using NIRS INVOS 5100 (Medtronic, Minneapolis, MN, USA) in addition to routine monitoring of SpO_2_ and HR to guide supplemental oxygen delivery and respiratory/circulatory support in preterm neonates during the first 15 min after birth.

The primary aim is to increase survival without cerebral injury at discharge or term age. The secondary aim is to assess neonatal morbidity until discharge or term age.

We hypothesize that supplemental oxygen support and respiratory/circulatory support guided by crSO_2_ and SpO_2_/HR monitoring during the first 15 min after birth will increase survival without cerebral injury and reduce morbidities in preterm neonates of less than 32 weeks of gestation.

## Methods

### Design

**Fig. 1:**
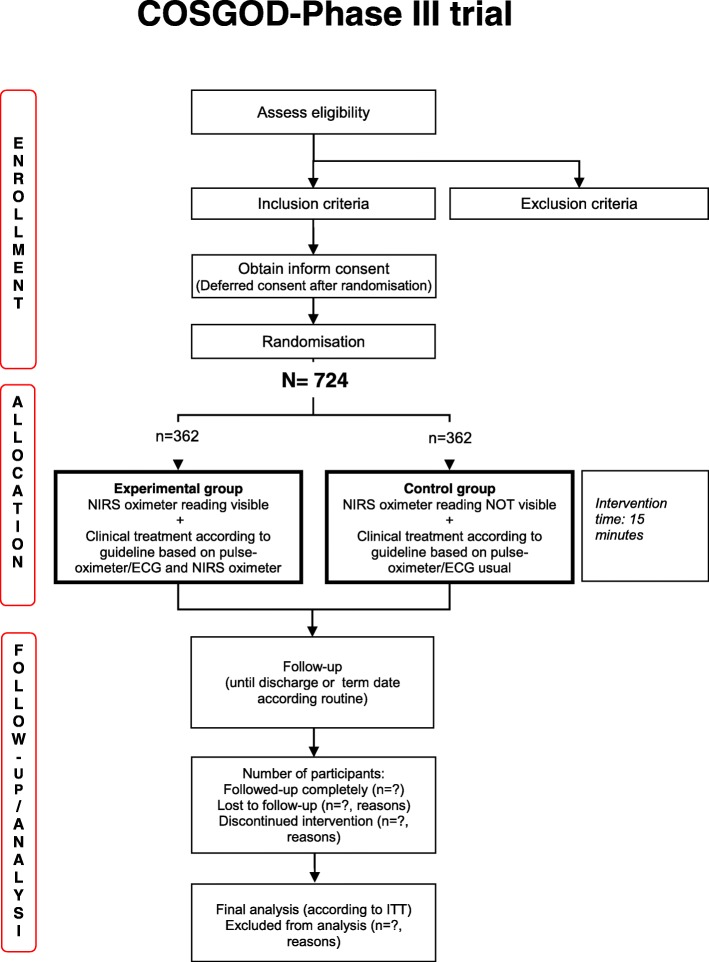
Trial flow chart. Abbreviations: *ECG* electrocardiogram, *ITT* intention-to-treat, *NIRS* near-infrared spectroscopy

**Fig. 2:**
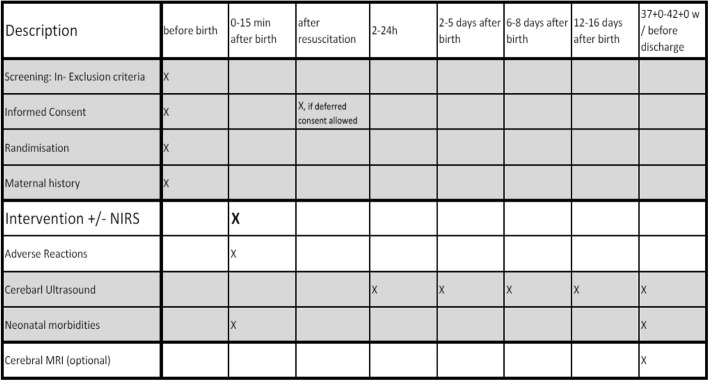
Schedule of enrolment, intervention and assessments. Abbreviations: *MRI* magnetic resonance imaging, *NIRS* near-infrared spectroscopy

The present trial is an investigator-initiated, randomized, multi-center, multi-national, clinical trial that will enroll 362 preterm neonates of less than 32 weeks of gestation in the study group and 362 preterm neonates of less than 32 weeks of gestation in the control group (Figs. 1 and 2). The trial is carried out in accordance with the Declaration of Helsinki in its latest form and the “International Conference of Harmonization – Good Clinical Practice” (ICH GCP) guidelines.

### Patients

Preterm neonates of less than 32 weeks of gestation are eligible for the study. Further inclusion criteria are the decision to provide full life support, written informed consent, and the application of NIRS sensors within 3 min after birth. Exclusion criteria are decision not to provide full life support, no written informed consent, or severe congenital malformation of brain, heart, lung, or prenatal cerebral injury or a combination of these.

### Sample size and statistical analyses

According to data of two European centers (Medical University of Graz and Erasmus Medical Center in Rotterdam) and one Canadian center (Royal Alexandra Hospital in Edmonton), the percentage of neonates who survive without cerebral injury ranges from 56% to 77% and the overall percentage is 65%. Given an increase of neonates who survive without cerebral injury from 65% to 75%, 329 neonates in each group are required to detect this difference with a two-group chi-squared test with a 0.05 two-sided significance level and a power of 80%. Given a dropout rate of 10%, a total of 724 neonates will be enrolled.

Demographic and baseline data for the infants will be compared between groups (study group versus control group) using the chi-squared test or Fisher’s exact test in the case of categorical variables and using the *t* test or Mann–Whitney *U* test in the case of continuous variables. To investigate the primary hypothesis whether the frequency of cerebral injury in preterm neonates of less than 32 weeks of gestation will be different in the two groups, a chi-squared test will be performed. Secondary outcome parameters including mortality and neonatal morbidities (cerebral injury, culture-proven early-onset infection/sepsis, necrotizing enterocolitis, bronchopulmonary dysplasia, retinopathy of prematurity, and persistent ductus arteriosus receiving intervention) will be compared between groups by using the chi-squared test or Fisher’s exact test. Exploratory outcomes (need for respiratory support, intubation, and medications during resuscitation, need for mechanical ventilation and treatment with catecholamines on the first day after birth) will be compared between groups by using the chi-squared test or Fisher’s exact test; trends of monitoring parameters (SpO_2_, HR, and NIRS parameters) during the first 15 min after birth will be analyzed by using linear mixed models. With these linear mixed models, differences in the course of the monitored parameters between groups (study group versus control group) will be analyzed. Baseline characteristics, which will show significant differences between groups, will also be included in the linear mixed models.

### Randomization

Neonates will be randomly assigned before birth to either the study group or the control group by using the web-based randomization service (“Randomizer for Clinical Trials”), developed at the Institute for Medical Informatics, Statistics and Documentation, Medical University of Graz (https://www.randomizer.at/random/). The ratio of allocation is 1:1. Patients will be stratified according to trial site. In case of multiple births, only the first infant will be randomly assigned.

### Informed consent procedure

Parents of potential participants will be invited to enroll their preterm neonates before delivery or, if permitted by local regulations, after delivery and resuscitation (deferred consent).

### Groups

In the study group, supplemental oxygen support and respiratory/circulatory support are guided by crSO_2_ and SpO_2_/HR monitoring. In the control group, supplemental oxygen support and respiratory/circulatory support are guided by SpO_2_/HR monitoring according routine resuscitation management; the resuscitation team is blinded to the crSO_2_ monitoring.

### Duration of interventions

Monitoring and clinical interventions start within 3 min after birth and last for 15 min after birth.

### Duration of follow-up

Each neonate will be followed until discharge or until term date (37–42 weeks of gestation).

Cerebral ultrasound will be performed at 2–24 h (optional), 2–5 days, 6–8 days, 12–16 days, before discharge/37–42 weeks of gestation and MRI (optional) before discharge, or at 37–42 weeks of gestation.

### Monitoring

The antepartum medical history and birth history will be collected. Gestational age, birth weight, gender, pH of umbilical artery, and Apgar score will be documented for each neonate. Continuous positive airway pressure and positive pressure ventilation with a face mask, if necessary, will be performed.

Monitoring during the first 15 min after birth consists of (i) pulse oximetry for SpO_2_ and HR as routine non-invasive monitoring, (ii) ECG for HR as routine non-invasive monitoring, and (iii) NIRS measurements. NIRS measurements will be blinded in the control group.

For NIRS measurements, the Invos™ Cerebral/Somatic Oximeter monitor (Medtronic) with the neonatal sensor will be used.

Immediately after birth, when the neonate is brought to the resuscitation area, the cerebral NIRS sensor will be placed on the left forehead and fixed with a continuous positive airway pressure cap or elastic bandage on the left forehead. ECG electrodes will be applied to the chest for HR monitoring. One pulse-oximetry sensor will be applied to the right palm or wrist for monitoring pre-ductal SpO_2_ and HR.

### Interventions

Resuscitation will be conducted in accordance with the “Consensus Guidelines” on the management of neonatal respiratory distress syndrome [28, 29], but cerebral oxygen saturation targeting and resuscitation will be at the discretion of the clinical team.

### Control group

**Fig. 3:**
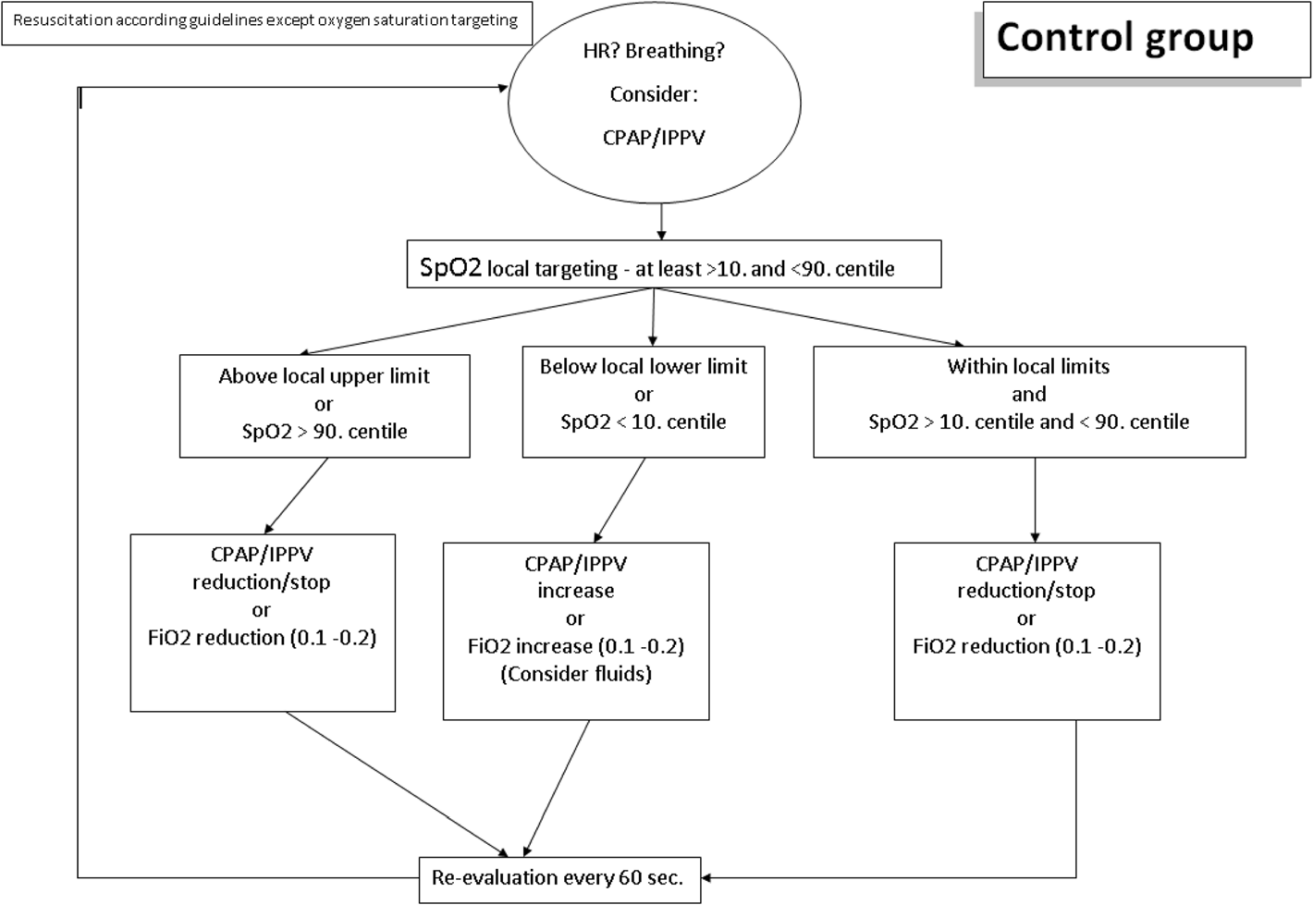
Intervention - Control group. Abbreviations: *CPAP* continuous positive airway pressure, *FiO*_*2*_ fraction of inspired oxygen, *HR* heart rate, *IPPV* intermittent positive-pressure ventilation, *SpO*_*2*_ arterial oxygen saturation

Depending on the infants’ breathing efforts and HR, the SpO_2_ targeting is conducted in accordance with “Local Guidelines” and “Resuscitation Guidelines” [28, 29] but at least between the 10th and 90th centiles [30] (Fig. 3). If SpO_2_ remains less than the 10th centile or below the local lower limit, respiratory support will be increased/started or fraction of inspired oxygen (FiO_2_) will be increased by 10–20% every 60 s. Respiratory support will be reduced/stopped or FiO_2_ will be reduced by 10–20% if SpO_2_ remains stable more than the 10th centile [30] or above the local lower limit for more than 60 s or if SpO_2_ is more than the 90th centile [30] or above the local upper limit. The clinical team will be blinded to the crSO_2_ monitoring.

### Study group

**Fig. 4:**
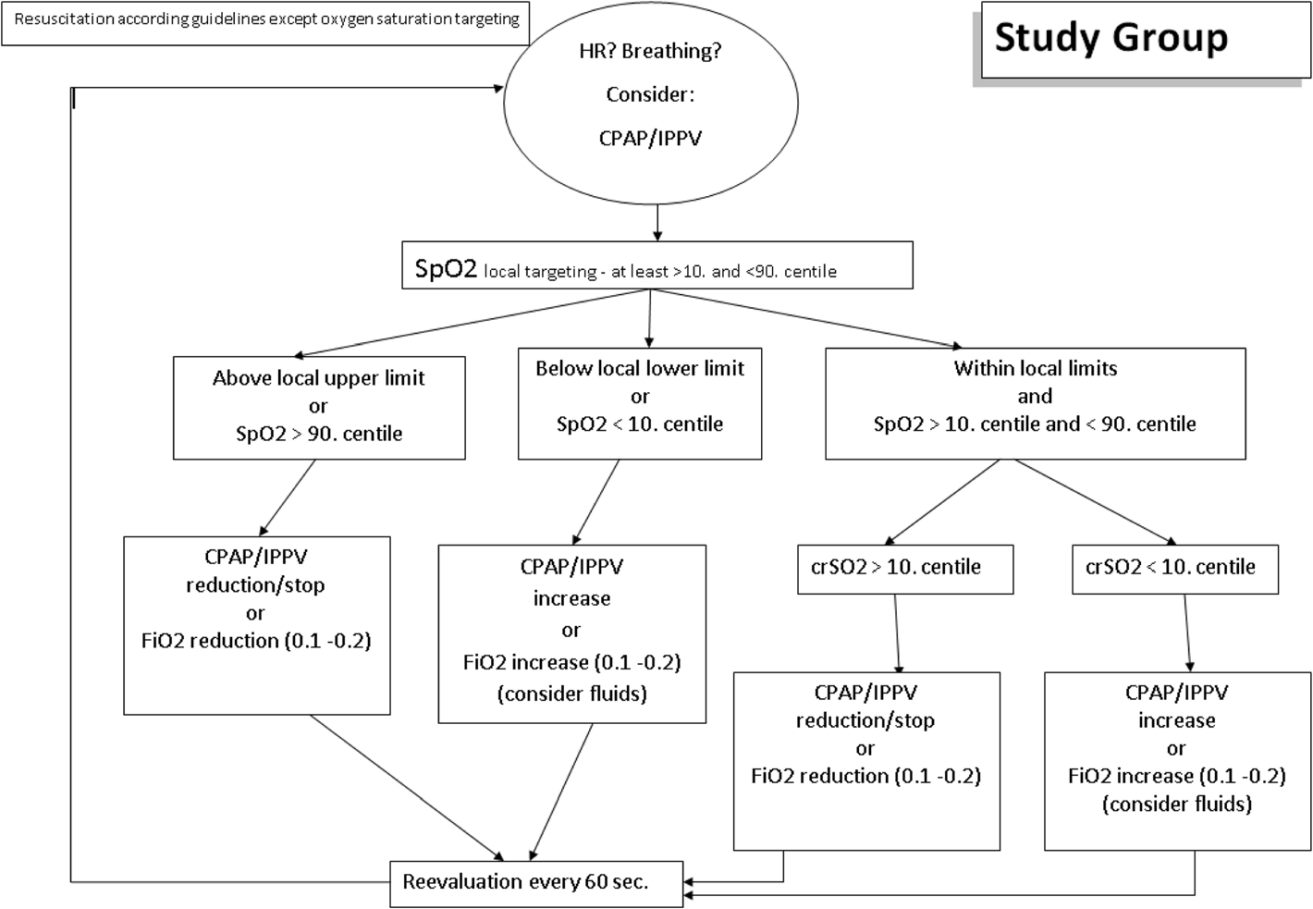
Intervention - Study group. Abbreviations: *CPAP* continuous positive airway pressure, *FiO*_*2*_ fraction of inspired oxygen, *HR* heart rate, *IPPV* intermittent positive-pressure ventilation, *SpO*_*2*_ arterial oxygen saturation

**Fig. 5:**
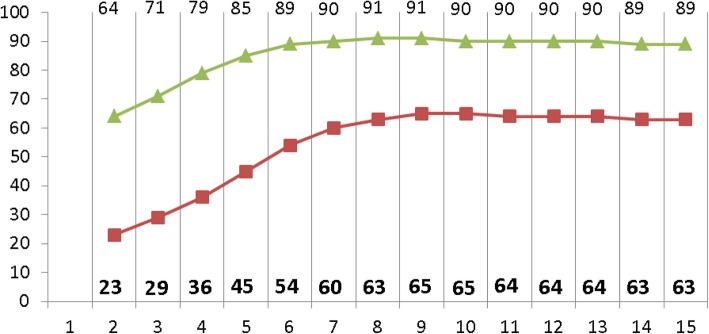
10th and 90th centiles of cerebral tissue oxygen saturation (crSO_2_) in each minute after birth (according to Table 1 “All neonates” published in Pichler et al. [31])

In the study group, the crSO_2_ monitoring is visible to the clinical team (Fig. 4). If SpO_2_ is within local limits and at least between the 10th and 90th centiles, the crSO_2_ value will be considered. If crSO_2_ is less than the 10th centile (Fig. 5) [31], respiratory support will be started or increased or oxygen support will be increased every 60 s by 10–20%. In case of history of volume loss and clinical signs of volume loss, an administration of intravenous fluid (10 mL/kg) can be considered [28, 29]. Respiratory support will be reduced/stopped or FiO_2_ will be reduced by 10–20% if crSO_2_ remains stable more than the 10th centile [31] for more than 60 s or if crSO_2_ is more than the 90th centile [31].

### Outcome measure

The primary outcome measure is survival without cerebral injury defined as any grade of IVH or cystic PVL*.* The secondary outcome measures include mortality and neonatal morbidities (cerebral injury, culture-proven early-onset infection/sepsis, necrotizing enterocolitis, bronchopulmonary dysplasia, retinopathy of prematurity, and persistent ductus arteriosus receiving intervention)*.* The exploratory outcome measures are need for respiratory support, intubation, and medications during resuscitation; need for mechanical ventilation and treatment with catecholamines on the first day after birth; and monitoring parameters (SpO_2_, HR, and NIRS parameters) during the first 15 min after birth.

### Outcome assessment tools

All-cause mortality will be recorded. Cerebral injury will be assessed by cerebral ultrasound at 2–24 h (optional), 2–5 days, 6–8 days, 12–16 days, before discharge/37–42 weeks and optionally cerebral injury will be assessed by cerebral MRI, if performed routinely—mainly without sedation during postprandial sleep—before discharge or term age (37–42 weeks of gestation). Case history will be assessed until discharge or term age, depending on what comes first.

### Blinding

Owing to the nature of the trial, the intervention cannot be blinded for the clinical staff and the parents. However, blinding will be used in some other aspect of the trial. The allocation sequence will be concealed.

### Data management

Source data will be registered in the participant’s medical records/case report form (CRF) and the electronic CRF (eCRF). A common web-based eCRF will be used and entered in a central database (Medical Informatics, Statistics and Documentation, Medical University of Graz). Data entry into the central database is the responsibility of the investigators. After establishment of a “clean file”, the database will be locked, and data will be stored for statistical analysis at the Institute for Medical Informatics, Statistics and Documentation, Medical University of Graz.

Trial data will be handled in accordance with regulations of the data protection agency in the respective countries. After completion of statistical data analysis, data will be pseudo-anonymized and deposited at the Medical University of Graz. After the end of trial, the data will be archived for 15 years in accordance with GCP guidelines. At each trial site, the data flow will be monitored in accordance with the GCP principles by a locally appointed external monitoring committee.

### Safety

The preterm patient population is a very vulnerable and often seriously ill group. Most adverse events may be of a serious nature with or without the COSGOD trial intervention, and both groups are expected to have a very high proportion of serious adverse events (SAEs). It is therefore not possible, or meaningful, to record and report all adverse events. The SAE to be reported is mortality. Serious adverse reaction and suspected unexpected serious adverse reactions (SUSARs) will be reported to ethic committees and authorities.

An independent safety committee will perform a first monitoring after the inclusion of 20% of the neonates to evaluate the risk of SAEs and efficacy (primary and secondary outcome parameters) of the intervention with “certain” or “probably/likely” relationship with the cerebral NIRS oximeter or the application of the treatment guideline. The participants will be insured in accordance with existing legislation of their respective country.

### Ethical considerations

The COSGOD trial will start at the different centers randomly assigning participants after approvals from the relevant ethics committees and authorities have been received. All parents will receive written and oral information about the trial before they are asked for their written consent. They will enroll their newborn infant in the trial only by their own free will and can withdraw their consent for participation at any time. If a parent wishes to withdraw the consent for participation, the patient will receive treatment in accordance with the respective hospital’s standard procedures. The trial will be conducted in compliance with the guidelines of the Declaration of Helsinki in its latest form and the ICH GCP guidelines.

In case of modifications in the study protocol that are not merely of a formal nature but contain changes pertinent to the study participants, a renewed vote of the ethics committee will be obtained. If applicable, the patients/parents will be informed in the patient information and consent form about changes in the terms and conditions of the trial.

## Discussion

The primary aim of the present clinical trial is to increase survival without cerebral injury in preterm neonates by monitoring crSO_2_ in addition to routine monitoring of SpO_2_ and HR to guide supplemental oxygen delivery, respiratory support, or circulatory support (fluid bolus) (or a combination of these) in preterm neonates during the first 15 min after birth. In the pilot study [23], we demonstrated feasibility of NIRS measurements even in extremely-low-birth-weight infants. Burden of cerebral hypoxia was reduced without any SUSARs; in addition, a trend toward reduction of cerebral injury was observed. The SafeBoosC phase II randomized clinical trial recently demonstrated that the combination of crSO_2_ measurement and a treatment guideline could reduce the burden of cerebral hypoxia in preterm neonates with less than 28 weeks of gestation during the first three days after birth [32]. In this study, a trend toward reduction of cerebral injury was also observed. Both, COSGOD phase I/II and SafeBoosC phase II had trends to lower mortality or cerebral injury (or both), suggesting a positive effect on short-term outcome; however, neither study was powered for cerebral injury [23, 32].

Since pulse oximetry and ECG are recommended during immediate transition and resuscitation when monitoring is performed [28, 29], both monitoring methods will be performed in all neonates. SpO_2_ will be kept within recommended/local limits in all neonates (at least between the 10th and 90th centiles of published reference ranges) [30]. Therefore, in the study group and in the control group, hypoxia defined as SpO_2_ below the lower limit and hyperoxia defined as SpO_2_ above the upper limit should be avoided.

Monitoring of crSO_2_ would suggest increased respiratory support and increased use of supplemental oxygen in case of low cerebral oxygenation. In the COSGOD phase I/II trial, the supplemental oxygen support was lower in the NIRS-visible group in the first minutes [23]. For COSGOD III, according to ICH GCP, the research and clinical team involved in conducting this trial should be qualified by education, training, and experience to perform NIRS measurements and interventions.

Owing to the short intervention period, irritation of the skin caused by NIRS sensors will be unlikely. Moreover, this has not been observed in other studies using NIRS during immediate transition [2, 11]. Risks related to the manipulation of the patient during positioning and re-positioning of the NIRS sensors will be minimized by experience in use of NIRS. Neonates will always be continuously monitored and observed by the resuscitation team.

In conclusion, additional monitoring of crSO_2_ and dedicated interventions are feasible during immediate transition and resuscitation and reduce the burden of cerebral hypoxia. The present trial will examine whether monitoring of crSO_2_ and dedicated interventions will improve survival without cerebral injury.

## Trial status

Recruitment started in September 2018 and is expected to be completed at the end of 2020.

## Abbreviations

CBF: Cerebral blood flow
CRF: Case report form
crSO_2_: Cerebral tissue oxygen saturation
ECG: Electrocardiogram
eCRF: Electronic case report form
FiO_2_: Fraction of inspired oxygen
GCP: Good Clinical Practice
HR: Heart rate
IVH: Intra-ventricular hemorrhage
MRI: Magnetic resonance imaging
NIRS: Near-infrared spectroscopy
PVL: Periventricular leukomalacia
SAE: Serious adverse event
SpO_2_: Arterial oxygen saturation
SUSAR: Suspected unexpected serious adverse reaction
